# Case Report: Clinical and Pharmacokinetic Profile of Lithium Monotherapy in Exclusive Breastfeeding. A Follow-Up Case Series

**DOI:** 10.3389/fphar.2021.647414

**Published:** 2021-06-24

**Authors:** Maria Luisa Imaz, Dolors Soy, Mercé Torra, Llüisa García-Esteve, Cristina Soler, Rocio Martin-Santos

**Affiliations:** ^1^ Perinatal Mental Health Clinic-BCN Unit, Department of Psychiatry and Psychology, Hospital Clínic, Department of Medicine, Institute of Neuroscience, University of Barcelona (UB), Institut D’Investigacions Biomèdiques August Pi I Sunyer (IDIBAPS), and Centro de Investigación Biomédica en Red en Salud Mental (CIBERSAM), Barcelona, Spain; ^2^ Division of Medicines, Pharmacy Service, Hospital Clínic, IDIBAPS, UB, Barcelona, Spain; ^3^ Pharmacology and Toxicology Laboratory, Biochemistry and Molecular Genetics Service, Biomedical Diagnostic Center (CBD), Hospital Clínic, IDIBAPS, and Department of Medicine, UB, Barcelona, Spain; ^4^ Department of Neonatology, Institut Clínic de Ginecologia, Obstetrícia i Neonatologia (ICGON), Hospital Clínic, Barcelona, Spain

**Keywords:** bipolar disorder, lithium, lactation, case report, pharmacokinetics, exclusive maternal breastfeeding, delivery, nursing infant

## Abstract

**Background:** Most guidelines advise that women taking lithium should not breastfeed. The variation in transfer is just one reason behind this advice.

**Objectives:** To present clinical and pharmacokinetic data of nine mother–infant pairs exposed to lithium monotherapy during late pregnancy and exclusive breastfeeding at the Perinatal Psychiatric Unit (2006–2018).

**Methods:** We obtained sociodemographic data, medical risk factors, obstetric variables, and family and personal psychiatric history by semi-structured interview, and assessed maternal psychopathology with the Hamilton Depression Rating Scale and Young Mania Rating Scale. A senior neonatologist reviewed neonatal outcomes at birth using the Peripartum Events Scale. Paired maternal and cord blood and infant venous blood samples were collected. During the breastfeeding period, we monitored serum lithium and creatinine concentrations in mother–infant pairs at delivery, and at days 1–5, 7–11, 30, and 60 postpartum, and monthly until 6-months.

**Results:** Lithium equilibrated completely across the placenta [1.13 (0.10), range (1.02–1.30)]. No women presented symptoms of postpartum lithium intoxication, two of the neonates presented transient hypotonia (22%). Lithium exposure was significantly less during breastfeeding than during late pregnancy, and serum lithium concentrations decreased up to 44% overtime from delivery to the first-month, and up to 60% to the third-month postpartum. There was no growth or developmental delay in the follow-up period. One woman had a manic episode with psychotic features at 45 days postpartum.

**Conclusions:** In carefully selected women with bipolar disorder, lithium therapy when breastfeeding can be an appropriate option if coupled with close monitoring of the mother-infant pair.

## Introduction

Lithium is an effective maintenance treatment for some women with bipolar disorder (Yatham et al., 2018), but there are legitimate concerns about its use during pregnancy and breastfeeding. However, women with bipolar disorder are at high risk of symptom relapse during the perinatal period (Munk-Olsen et al., 2009; Viguera et al., 2011; Wesseloo et al., 2016), and those treated with lithium have a significantly lower rate of relapse during this period (Bergink et al., 2015).

Lithium, a monovalent cation that is absorbed rapidly after oral intake, is not metabolized or bound to proteins and is eliminated almost exclusively via the kidneys. Lithium elimination half-life is about 18–24 h in healthy young women. During pregnancy, serum lithium concentrations decline in relation to the increase in intravascular volume and the glomerular filtration rate (GFR) (Grandjean and Aubry, 2009). Lithium shows complete placental passage and equilibrates between the maternal and fetal circulation across a wide range of maternal concentrations (0.2–2.6 mEq/L) (Newport et al., 2005). Indeed, it has been suggested that the accumulation of lithium in fetal serum may be associated with an increased rate of neonatal complications, sometimes referred to as “floppy baby syndrome” (Kozma, 2005). An association has been observed between high infant lithium concentrations (>0.64 mEq/L), lower 1-min Apgar scores, longer hospital stays, and higher rates of central nervous system and neuromuscular complications (Newport et al., 2005). The regular measurements of the serum lithium concentration are needed perinatally to ensure that it remains within the therapeutic range, and to minimize the risk of both maternal and neonatal complications (Malhi et al., 2017; Wesseloo et al., 2017; Westin et al., 2017). Women are advised to suspend lithium treatment at the onset of labor or for 24–48 h before a scheduled cesarean section (Newport et al., 2005).

During the postpartum period, the maternal serum lithium concentration gradually returns to its preconception level, potentially risking lithium intoxication if women had increased their dose during pregnancy. Lithium is also excreted into breastmilk. Lithium transfer from milk to the infant shows a high variability (20–100%) (Newmark et al., 2019). In infants of mothers receiving lithium maintenance treatment in late pregnancy, and who choose to perform exclusive breastfeeding, two simultaneous phenomena are known to occur in the first weeks of life. On the one hand, it is the elimination of lithium transferred through the placenta, and on the other, is the absorption of lithium transferred through breast milk. Moreover, in front of the infant immaturity of renal function and the increased amount of milk consumption, lithium accumulation and potential intoxication may occur.

Despite the Food and Drug Administration (Food and Drug Administration, 2005) recommending clinical lactation studies for psychopharmaceuticals, data remains limited and uncertainty persists regarding the safe use of lithium during breastfeeding. (The American Academy of Pediatrics (AAP), 2012; Sachs, 2013) has been suggested that lithium can be continued during breastfeeding, provided there is careful monitoring of serum lithium concentrations, as well as renal and thyroid function, in the infant (Viguera et al., 2007). Others recommend only standard pediatric care, including monitoring weight and feeding in the first 2 weeks postpartum (Bogen et al., 2012). However, most international guidelines and some perinatal psychiatrists believe that lithium exposure *via* breast milk could be dangerous and recommend using infant formula (Malhi et al., 2017; Galbally et al., 2018).

Breastfeeding has many important health advantages for both mothers and their children (Rollins et al., 2016; Vitora et al., 2016). The American Academy of Pediatrics (AAP), 2012 recommend exclusive breastfeeding for the first 6 months of life whenever possible, before combining it with complementary foods until the infant is 1–2 years old. Two recent systematic reviews of clinical studies into lithium use during breastfeeding found limited evidence about whether one should initiate, maintain, or discontinue lithium during breastfeeding (Imaz et al., 2019; Newmark et al., 2019).

The aim of this study was to examine serum lithium concentrations in mothers and their exclusively breastfed term infants from delivery.

## Method

### Subjects

We included women with bipolar disorder (DSM-IV or DSM-V) treated with lithium monotherapy, who were clinically stable at least during late pregnancy, and who chose to breastfeed exclusively (N = 9). The patients attended the Perinatal Psychiatry Clinic-BCN Unit between 2006–2018. All gave their written informed consent for the use of paired data from themselves and their infants.

All women were informed of the known risks associated with fetal and infant exposure to lithium during pregnancy and breastfeeding, as well as the risks associated with discontinuing lithium treatment or suffering untreated maternal bipolar disorder, based on current evidence. All women were treated with lithium carbonate, twice at day. The lithium dose was adjusted according to clinical status and serum lithium concentrations during pregnancy. At 33–35 weeks of pregnancy, the women and their partners devised a birth and breastfeeding plan with a psychiatrist. Women were empirically advised to suspend lithium treatment at the onset of labor in the event of spontaneous deliveries or for 12 h before a scheduled cesarean section or induction. Lithium was restarted 6–12 h after delivery.

### Assessments

#### Mothers

During the first visit during pregnancy, all women completed a semi-structured interview that included questions on their sociodemographic characteristics, medical risk factors, parity, past obstetric complications, and index pregnancy planning. We also recorded any personal and family psychiatric history, substance use, and type of relationship with their partner. A senior psychiatrist administered the validated Spanish versions of the 17-item Hamilton Depression Rating Scale with the Atypical Depression Supplement (Bobes et al., 2003), the Young Mania Rating Scale (Colom et al., 2002), and the Functioning Assessment Short-Test (Rosa et al., 2007) at baseline and follow-up visits. We also reviewed obstetric records to collect information on obstetric risk factors, indication for admission for labor and delivery, method of delivery, and delivery complications. Women were asked about the level of satisfaction with lithium treatment during exclusive breastfeeding period using a visual analog scale (from very poor to very high) at each follow-up visit.

#### Neonates and Infants

A senior neonatologist reviewed delivery records to collect information on the newborns, from the physical examination performed by the neonatologist at birth (12–24 h of life), admission into a neonatal intensive care unit, and data on neonatal signs obtained with the infant subscale of the Peripartum Events Scale (O’Hara et al., 1986). After hospital discharge infants were evaluated by their pediatrician in accordance with the current standardized clinical protocol (Public Health Agency of Catalonia, 2019).

#### Blood Sample Collection

During pregnancy routine tests were performed to monitor maternal complete blood counts, glucose levels, and electrolytes; kidney, liver, and thyroid function; serum lithium concentrations; and urinalysis (5 ml) to exclude substance use. Blood samples (10 ml) were collected in the morning before the first daily dose of lithium, at 10–14 h under steady-state conditions.

At delivery, maternal and cord blood samples (10 ml) were collected simultaneously to record serum lithium concentration. At 48–72 h postpartum, a pediatric nurse performed a neonatal screening test to identify any metabolic or endocrine diseases (Public Health Agency of Catalonia, 2013). During postpartum, we monitored lithium and creatinine serum concentrations simultaneously in the mother–infant pairs at 1–5 and 7–11 days, at 1 month, and monthly thereafter while breastfeeding. Two pediatric phlebotomists collected 5 ml of venous blood from the mothers and 2 ml from the infants before the first daily maternal lithium dose. Infant blood analysis was stopped if lithium concentrations in the nursing infant were below the limit of quantification in two consecutive samples, and/or the nursing infant combined with complementary foods, and/or the mother changed to bottle feeding. We also collected a urine sample (5 ml) from mothers to monitor substance use.

#### Lithium Serum Analysis

For serum lithium analysis, we collected maternal venous blood, cord blood, and neonate/infant venous blood in BD Vacutainer^®^ No-Additive Z Plus tubes (BD Diagnostics, Preanalytical Systems, NJ-07417). Lithium concentrations were determined by an AVL 9180 electrolyte analyzer based on the ion-selective electrode measurement principle (Roche Diagnostics, IN-46256). Two-point calibration was performed every 4 h. The detection limit was 0.10 mEq/L, and the limit of quantification was 0.20 mEq/L. The within- and between-day precisions, expressed as coefficients of variation, were 0.97–4.1% and 1.3–6.4%, respectively. The therapeutical range of lithium has been stablized at 0.5–1.2 mEq/L. The toxic concentration for lithium is ≥ 1.5 mEq/L (Hiemke et al., 2018).

#### Other Serum Analyses

Serum creatinine levels were measured using the Jaffé method (Modular P, Roche Diagnostics) for traceable measurements, using isotope dilution mass spectrometry. The within- and between-day precisions, expressed as coefficients of variation, were 1.5 and 2.5%, respectively. The modified Schwartz formula (Schwartz et al., 2009), which uses serum creatinine (Scr), height, and an empirical constant [(Kxheight)/Scr]) was used to estimate the neonate/infant GFR. Neonatal thyroid stimulating hormone (TSH) levels were analyzed using the 1,235 AutoDelfia^®^ automatic immunoassay system that used dry blood samples on filter paper (PerkinElmer, Inc.).

### Statistical Analysis

All data were analyzed with SPSSv25. A descriptive analysis was performed to characterize the sample and the placental passage of lithium, using the mean and standard deviation (range) for quantitative variables. The ratio of the lithium concentration in umbilical cord to that in maternal plasma was calculated for each maternal–infant pair as an index of the lithium placental passage. In addition, we calculated the within-subject change in lithium serum concentrations for infants from baseline to each assessment point, reporting as mean (standard deviation) and 95% confidence intervals (CIs). We expressed the difference of means results as a percentage of change. We also used within-subject means to examine Pearson correlations between maternal and infant serum lithium concentrations.

## Results

### Characteristics of the Sample

Table 1 shows the sociodemographic, medical, and obstetric characteristics of each case (N = 9). All women were receiving lithium monotherapy in the third trimester of pregnancy and all were clinically stable. Seven had been taking lithium throughout pregnancy and two had started it during pregnancy, at gestational weeks 25 and 36. None were taking medications known to interact with lithium (Table 1). Seven women had mean lithium concentrations within the therapeutic range when sampled at steady-state at their most recent prenatal visit 0.79 ± 0.19 (0.50–1.10) mEq/L. Two women did not accept dose adjustments because they were stable during pregnancy and had a history of maintaining lower serum lithium levels without relapse (Cases 4 and 6).

**TABLE 1:** Maternal characteristics, obstetric outcomes, and treatment during pregnancy.

	CASE-1	CASE-2	CASE-3	CASE-4	CASE-5	CASE-6	CASE-7	CASE-8	CASE-9
Planned pregnancy	Planned happy	Planned happy	Unplanned happy	Planned happy	Accident	Unplanned happy	Planned happy	Unplanned happy	Planned happy
Parity	Primiparous	Multiparous	Primiparous	Primiparous	Primiparous	Primiparous	Primiparous	Primiparous	Multiparous
Medical risk factors^a^	None	None	None	None	None	None	None	None	None
Obstetric risk factors	GD^b^	Mild PE^c^ at D	GD^b^/PE wk 38.4	None	None	None	None	None	None
Indication to labor and delivery	None	None	PE	None	None	PROM >12 h	None	None	None
Method of delivery	Vaginal	Vaginal	C-section	Vaginal	Vaginal	Vaginal induction	Vaginal	Vaginal induction	Elective C-Section
Delivery complications	None	None	None	None	None	None	None	None	None
Personal psychiatric diagnosis	BD II	BD nos	BD I	BD I	BD I	BD I	BD I	BD I	BD I
Family psychiatric diagnosis	None	None	None	None	Mother BD I	None	Paternal grandfather BD I	None	None
Lithium (Li) dose (mg/day)	800	800	800	800	800	800	1,200	1,000	1,200–1,600
Li treatment duration	wk25-D	wk0-D	wk0-D	wk0-D	wk36-D	wk8-D	wk0-D	wk8-D	wk0-D
Other medication	FXT wk14-wk28 DZP 5 ad lib	None	LOR 0.5 ad lib ASA 100	CZP 0.5 ad lib	None	ASA 100	None	None	None
Urine drug test^d^	Negative	Negative	Negative	Negative	Negative	Negative	Negative	Negative	Negative

Abbreviations: ASA, acetylsalicylic acid; B, baseline (pregnancy first visit); BD, bipolar disorder; C-section, cesarean section; CZP, clonazepam; D, delivery; DZP, diazepam; FXT, fluoxetine; GD, gestational diabetes treated with diet; NA, not available; PROM, premature rupture of membranes; PE, preeclampsia; wk, week.

a Medical risk factors including hypertension, heart disease, endocrine disease, kidney disease, pulmonary disease, gastrointestinal disease, seizure disorder, anemia (Hgb <9.0), extremes of pre-pregnant weight (<45 or >90 kg).

b Gestational diabetes treated with diet.

c Protein alteration without clinical symptomatology.

d Urine drug test included cotinine, benzodiazepines, cannabis, heroin, methadone, cocaine, amphetamine.

### Maternal and Neonatal Serum Lithium Concentrations at Delivery

Supplementary Table S1 details the serum lithium concentrations of mothers and infants from delivery onward. At delivery, serum lithium concentrations were determined from nine maternal samples and eight umbilical cord samples and were 0.41 ± 0.15 (0.19–0.72) mEq/L and 0.44 ± 0.16 (0.23–0.76) mEq/L, respectively. Umbilical cord lithium concentrations exceeded maternal concentrations in all paired analyses. Hematocrit levels at delivery were 36.66 ± 12% (19.60–65.00%) in mothers and 48.24 ± 9.89% (32.80–65.00%) in umbilical cords.

Seven of the nine mothers showed sub-therapeutic (<0.50 mEq/L) lithium concentrations of 0.34 ± 0.09 (0.19–0.43) mEq/L at delivery. The mean time from the last dose to delivery was 28.11 ± 14.59 (12–56) hours. Despite the lower serum lithium concentrations at delivery, the mean daily lithium dose in mothers was 955.56 ± 278.88 (600–1,600) mg/day and the mean infant–mother lithium ratio at delivery was 1.13 ± 0.10 (1.02–1.30). The mothers restarted lithium a mean of 16.33 ± 8.10 (6–31) hours after delivery. Despite the brief peripartum interruption in therapy, none of the women decompensated.

### Maternal Satisfaction

Seven women showed a very high level and two moderate level of satisfaction with lithium treatment during exclusive breastfeeding period.

### Neonatal Physical Examination at Birth and Neonatal Outcomes

All neonates were full-term, and their outcomes are presented in Table 2. Although there were three cases of fetal acidosis at delivery and two cases of transient hypotonia, there were no signs of lithium toxicity or of other adverse clinical events in any infants. We observed a kinking of the ductus in one neonate, which was had resolved by the 2-months follow-up echocardiogram. Another neonate presented an isolated low implantation of the ear auricle.

**TABLE 2:** Characteristics of neonates/infants exposed to lithium during late pregnancy and exclusive breastfeeding.

Sex	CASE-1 Female	CASE-2 Male	CASE-3 Female	CASE-4 Female	CASE-5 Male	CASE-6 Male	CASE-7 Male	CASE-8 Male	CASE-9 Female
Gestational age weeks + days	39 + 5	40 + 5	38 + 5	37 + 4	39 + 6	38 + 0	41 + 2	40 + 3	39 + 1
Weight at birth (gr)	3,450	3,520	2,965	3,430	3,324	3,200	3,690	4,100	4,400
Length cm	51.00	51.00	46.00	51.50	50.00	50.00	52.00	53.00	53.00
Head circumference cm	33.00	36.00	35.00	33.00	34.50	34.50	37.00	35.00	37.50
Apgar 1/5/10 min	9/10/10	9/10/10	9/10/10	8/8/9	9/10/10	9/10/10	9/10/10	9/10/10	9/10/10
UA pH	7.24	7.24	7.27	7.22	7.04^a^	7.06^a^	7.27	7.25	7.02^a^
Neonatal TSH^b^	2.56	0.90	1.55	1.85	4.73	1.59	0.58	1.23	2.52
IS-PES neonatal sing (total score)^e^	0	0	0	0	0	0	0	0	^c^
Newborn physical examination (by systems)^d^	Normal	Dysmorphic auricle axial hypotonia	Non-restrictive kinking of the ductus arteriosus	Normal	Normal	Normal	Axial hypotonia	Normal	Systolic murmur I/VI
Weight at 48 h postpartum (gr)	3,195	3,215	2,770	3,200	3,035	3,080	3,270	3,770	3,970
Hospital stay (days)	3	2	4	3	2	2	2	2	3
Exclusive breastfeeding duration (days)	131	36	15	180	17	171	45	123	98
Change of feeding type	Mixed	Infant formula	Infant formula	Complementary	Mixed	Complementary	Infant formula	Complementary	Infant formula
Reasons for termination of breastfeeding	Return to work	Nipple anatomy insufficient breastmilk	Nipple anatomy slow weight gain	Return to work	Slow weight gain	Return to work	Maternal relapse	Infant age	Infant weight crisis during growth

Abbreviations: N = normal; NA = not available; UA pH = umbilical artery pH; TSH = thyroid stimulant hormone; IS-PES = Infant subscale of Peripartum Events Scale.

a Fetal acidosis.

b Neonatal TSH: neonatal screening at 48 h of life (mU/mL).

c Hyperbilirrubinemia.

d Newborn physical examination by systems: skin and lymphatics, head, eyes, ears, nose, mouth and throat, neck-thyroid (goiter), lungs/thorax, cardiovascular (cardiomegaly, bradycardia, systolic murmur); abdomen/hepatic (hepatomegaly, jaundice); neuromuscular (hypotonia, flaccidity, diminished deep tendon reflexes, poor suck, Moro reflexes; central nervous system (lethargy, depression); genitourinary renal (polyuria, diabetes insipidus); respiratory (apnea, cyanosis, labored breathing, need for intubation), ano-genital.

e IS-PES neonatal sign included 11 items: need for pH correction, volume correction, need for transfusion or plasma exchange, hypoglycaemia, hypocalcemia, hyperbilirrubinemia, treatment for sepsis, meconium aspiration pneumonitis, other serious event, special care admission and treatment to alleviate distress.

### Maternal and Infant Serum Lithium Concentrations During Breastfeeding

Infants were exclusively breastfed for an average of 93 ± 65.26 (15–189) days (Table 2), and 53 samples were obtained from the nine mother–infant pairs between delivery (day 0) and 180 days postpartum (Supplementary Table S1). The maternal lithium dose averaged 987 ± 325 (400–1,200) mg/day, with a daily serum concentration of 0.76 ± 0.29 (0.41–1.31) mEq/L. No correlations were observed between maternal and infant serum lithium concentrations (data no shown).

**FIGURE 1:**
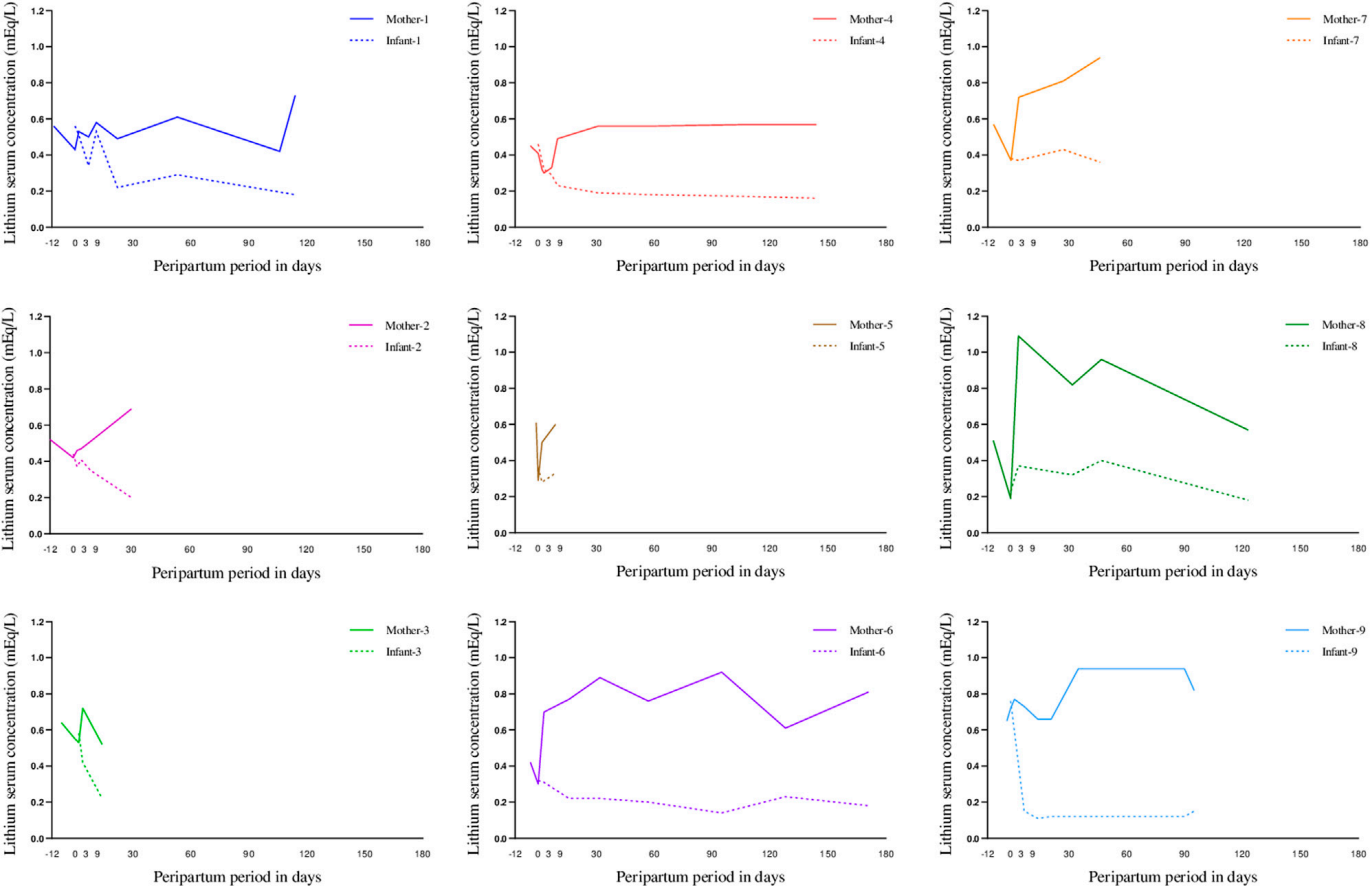
Mother and breastfeed infant lithium serum concentration at delivery and during exclusive maternal lactation case by case (case 1–9). “Day 0” represents delivery.

Table 3 shows how infant serum lithium concentrations declined over time from delivery to the third month postpartum, with levels decreasing in the first month of 43.80% (-38.45% to -43.88%), and at three months by 58.52% (95%CI: 38.22% to -78.90%) compared to delivery. The case-by-case data are available in Supplementary Table S1. Figure 1 shows the infant and maternal serum lithium concentrations during lactation.

**TABLE 3:** Estimated infant lithium serum concentration during exclusive breastfeeding.

Time postpartum	Number of serum lithium analyses available	Mean [Li] (mEq/L)	95% CI (mEq/L)	% Change from baseline	95% CI
Baseline (delivery)	8	0.43	0.32 to 0.55	-	
T1 (3 ± 2 days)	9	0.41	0.33 to 0.49	−6.31	−2.28 to −15.16
T2 (9 ± 2 days)	5	0.29	0.21 to 0.36	−33.45	−24.43 to −42.45
T3 (30 ± 5 days)	6	0.24	0.15 to 0.34	−43.80	−38.35 to −43.88
T4 (60 ± 5 days)	4	0.19	0.12 to 0.27	−54.99	−45.56 to −65.13
T5 (≥90 days)	5	0.18	0.15 to 0.20	−58.52	−38.33 to −78.90

Abbreviations: CI = Confidence interval; T = time.

Supplementary Table S1 shows that umbilical cord creatinine concentrations were similar to those of the mothers at delivery. By 1 week after delivery, all neonates showed a creatinine concentration over normal values (0.35–0.40) mg/dl. The mean neonatal eGFR at delivery was 36.70 ± 12.53 (24.87–64.53) mg/ml/1.73 m^2^, which continued to increase to >75 mg/ml/1.73 m^2^ with the age of the nursing infant. The mean neonatal TSH concentration was 1.95 ± 1.24 (0.58–4.73) mg/dl, falling within the normal range.

### Maternal and Infant Outcomes During Postpartum

At 45 days postpartum, one mother (case 7) experienced a manic episode with psychotic features despite a lithium concentration of 0.91 mEq/L. She was briefly hospitalized for 11 days and breastfeeding was stopped. Finally, no acute growth or developmental delays were reported by the pediatrician in any infant during the follow-up period (data not shown).

## Discussion

To our knowledge, this is the first study to have simultaneously examined serum lithium concentrations in both mother and infant from delivery through a period of exclusive breastfeeding. This is surprising given the lack of data supporting advice to stop or avoid lithium during breastfeeding.

A reason lithium is often discouraged is the possible risk of toxicity in nursing infants. Although umbilical cord lithium concentrations were slightly higher than maternal plasma concentrations at delivery, the serum concentration in nursing infants decreased during lactation, independently of the maternal serum lithium concentration. This may be because neonatal hematocrit levels were higher than maternal hematocrit levels at delivery (Lu et al., 1991). Moreover, we observed a smaller reduction in infant lithium concentrations (6%) in the first week of life compared with those that followed. It is likely that this reflects the physiological weight loss typically experienced by nursing infants in their first week (about 10%, mainly due to fluid loss), with lithium clearance being particularly sensitive to changes in fluid volume (Grandjean and Aubry, 2009).

Another reason for discouraging lithium use is the concern of adverse effects on kidney and thyroid function. However, despite elevated neonatal serum creatinine levels in the first few days of life, we observed no lithium-related nephrotoxicity in them while nursing. In full-term neonates, serum creatinine levels are normally elevated at birth, reflecting the mother’s kidney function due to fetal-maternal placental equilibration (usually 0.70 mg/dl), and this progressively decreases over several weeks to reflect the infant’s true kidney function (Mian and Schwartz, 2017). By contrast, the eGFR (mL/min/1.73 m^2^) is physiologically low in the first week of life (5–40 ml/min/1.73 m^2^) and continues to increase (up to 65 ml/min/1.73 m^2^ by age 2 months), reaching young adult levels (120–130 ml/min/1.73 m^2^) by approximately 2 years (Vieux et al., 2010). In all our cases, neonatal TSH concentrations were within the normal range at 48 h postpartum. We did not analyze TSH concentrations in nursing infants during the lactation period because exposure to lithium was less than that during the fetal period.

Sleep is often interrupted to feed and take care of infants during the first few months postpartum (Lee, 2000). Exclusive breastfeeding might worsen the disruption to sleep patterns (duration and fragmentation) (Doan et al., 2014), and this is an established trigger for relapses, particularly of mania, in women with bipolar disorder type I (Lewis et al., 2017). Women with bipolar disorder who report episodes of mania triggered by sleep loss are also twice as likely to experience an episode of postpartum psychosis (Lewis et al., 2018), with the period of highest risk of psychiatric readmission being 10–19 days postpartum (Munk-Olsen et al., 2009). A recent systematic review and meta-analysis revealed that postpartum relapse rates were significantly lower in women who used prophylactic medication during pregnancy than among those who received none (Wesseloo et al., 2016). In our cases, one woman (11%) had an episode of mania with psychotic features at 45 days postpartum despite a prenatal plan to minimize sleep disruption. This is less than described previously in the literature (Munk-Olsen et al., 2009; Wesseloo et al., 2016).

The study had several limitations. First, it was limited in both size and duration, but it benefited from including a carefully selected sample of clinically stable women with bipolar disorder who had received lithium monotherapy throughout late pregnancy and exclusive breastfeeding. Second, although the findings may not be generalizable to more heterogeneous populations of nursing women with bipolar disorder who are treated with lithium, the results contribute to the accumulating evidence helping clinicians and patients make informed decisions about lithium use during lactation. Third, we did not use a standardized neuropsychological assessment for the nursed infants during follow-up. Finally, we are aware of the limits of detectability of the assay used to measure lithium concentrations, especially for values below the limit of quantification.

## Conclusions

In carefully selected women with bipolar disorder who breastfeed exclusively, lithium can be considered an appropriate option if the infant is monitored closely. Special attention should be given to monitoring clinical features and lithium concentrations in mothers and infants at regular intervals (e.g., 1–5, 7–11, 30, and 60 days postpartum) or if clinical concerns arise. Collaborative studies are needed in larger cohorts to confirm our findings.

## Data Availability

The original contributions presented in the study are included in the article/Supplementary Material, further inquiries can be directed to the corresponding author.
