# Supplementary material for: Case Report: Clinical and Pharmacokinetic Profile of Lithium Monotherapy in Exclusive Breastfeeding. A Follow-Up Case Series
Source: Front Pharmacol. 2021 Jun 24;12:647414. doi: 10.3389/fphar.2021.647414 (PMC8264295; doi:10.3389/fphar.2021.647414)
Supplement: Supplementary file 1 [file Table1.DOCX]

| Supplementary Table 1. Maternal-Neonatal/Infant drug concentrations at delivery and during exclusive breastfeeding in lithium monotherapy | | | | | | | | | |  |
| --- | --- | --- | --- | --- | --- | --- | --- | --- | --- | --- |
| **Mother-infant**  **Case N** | **Infant age**  **at sampling**  **(days)** | **Maternal**  **Lithium dose**  **(mg/day)** | **Steady-state** | **[Li] serum**  **(mmol/L)** | | **[Li ]**  **Ratio I/P** | **Creatinine**  **mg/dL** | | **eGFR**  **mL/min/1.73m^2^**  **Neonate/Infant** | |
|  |  |  |  | **Mother**  **(P)** | **Neonate/Infant (I)** |  | **Mother** | **Neonate/Infant** |  |  |
| CASE-1 | **0** | **-** | **No** | **0.43** | **0.56** | **1.30** | **0.62** | **0.56** | **37.61** | |
|  | 2 | 800 | No | 0.53 | 0.51 | 0.96 | NA | 0.70 | - | |
|  | 7 | 800 | Yes | 0.50 | 0.34 | 0.68 | 0.73 | 0.11 | >75 | |
|  | 11 | 800 | Yes | 0.58 | 0.53 | 0.91 | 0.65 | 0.45 | - | |
|  | 22 | 800 | Yes | 0.49 | 0.22 | 0.44 | 0.70 | 0.42 | - | |
|  | 53 | 800 | Yes | 0.61 | 0.29 | 0.47 | 0.68 | 0.29 | >75 | |
|  | 106 | 800 | Yes | 0.42 | ^†^ | - | 0.73 | 0.39 | - | |
|  | 114 | 800 | Yes | 0.73 | 0.18 | 0.24 | 0.69 | 0.41 | - | |
| CASE -2 | **0** | **-** | **Yes** | **0.42** | **0.44** | **1.05** | **0.51** | **0.51** | **41.3** | |
|  | 2 | 800 | No | 0.46 | 0.37 | 0.80 | 0.47 | NA | - | |
|  | 4 | 800 | No | 0.47 | 0.41 | 0.87 | 0.51 | 0.67 | - | |
|  | 9 | 800 | Yes | 0.51 | 0.35 | 0.68 | 0.57 | 0.52 | - | |
|  | 30 | 800 | Yes | 0.69 | 0.20 | 0.28 | 0.62 | 0.40 | - | |
| CASE-3 | **0** | **-** | **No** | **0.55** | **NA** | **-** | **0.67** | **NA** | **NA** | |
|  | 1 | 800 | No | NA | 0.66 | - | NA | NA | - | |
|  | 2 | 800 | No | 0.53 | 0.58 | 1.09 | 0.70 | ^‡^ | - | |
|  | 4 | 800 | No | 0.72 | 0.42 | 0.58 | 0.61 | ^‡^ | - | |
|  | 14 | 800 | Yes | 0.52 | 0.22 | 0.42 | 0.74 | 0.62 | 31.31 | |
| CASE-4 | **0** | **-** | **No** | **0.41** | **0.46** | **1.12** | **0.63** | **0.59** | **36.05** | |
|  | 2 | 400 | No | 0.32 | NA | **-** | 0.57 | NA | - | |
|  | 3 | 400 | No | 0.30 | 0.32 | 1.06 | 0.61 | NA | - | |
|  | 7 | 400 | Yes | 0.33 | 0.29 | 0.87 | 0.74 | 0.40 | 53.17 | |
|  | 10 | 800 | No | 0.49 | 0.23 | 0.46 | 0.74 | ^‡^ | NA | |
|  | 31 | 800 | Yes | 0.56 | 0.19 | 0.33 | 0.94 | 0.31 | >75 | |
|  | 59 | 800 | Yes | 0.56 | 0.18 | 0.32 | 0.74 | 0.20 | >75 | |
|  | 109 | 800 | Yes | 0.57 | 0.17 | 0.29 | 0.75 | 0.21 | - | |
|  | 144 | 800 | Yes | 0.57 | 0.16 | 0.28 | 0.73 | 0.22 | >75 | |
| CASE-5 | **0** | **-** | **No** | **0.29** | **0.36** | **1.24** | **0.66** | **0.67** | **30.82** | |
|  | 2 | 800 | No | 0.50 | 0.28 | 0.56 | 0.72 | 0.55 | - | |
|  | 9 | 800 | Yes | 0.60 | 0.33 | 0.55 | 0.71 | ^‡^ | NA | |
| CASE-6 | **0** | **-** | **No** | **0.30** | **0.32** | **1.06** | **0.65** | **0.32** | **64.53** | |
|  | 3 | 1200 | No | 0.70 | 0.31 | 0.44 | 0.49 | 0.50 | 41.71 | |
|  | 16 | 1200 | Yes | 0.77 | 0.22 | 0.28 | 0.75 | 0.31 | - | |
|  | 32 | 1200 | Yes | 0.89 | 0.22 | 0.24 | 0.73 | 0.31 | - | |
|  | 57 | 1200 | Yes | 0.76 | 0.20 | 0.26 | 0.64 | 0.19 | >75 | |
|  | 95 | 1200 | Yes | 0.92 | 0.14 | 0.15 | 0.65 | NA | NA | |
|  | 128 | 1200 | Yes | 0.61 | 0.23 | 0.37 | 0.56 | 0.24 | >75 | |
|  | 171 | 1200 | Yes | 0.81 | 0.18 | 0.21 | 0.62 | 0.50 | 56.17 | |
| CASE-7 | **0** | **-** | **No** | **0.37** | **0.38** | **1.02** | **0.53** | **0.67** | **32.05** | |
|  | 4 | 1000 | No | 0.72 | 0.37 | 0.51 | 0.45 | 0.42 | 51.13 | |
|  | 27 | 1000 | Yes | 0.81 | 0.43 | 0.53 | 0.53 | ^‡^ | NA | |
|  | 46 | 1000 | Yes | 0.94 | 0.36 | 0.38 | 0.56 | 0.20 | >75 | |
| CASE-8 | **0** | **-** | **No** | **0.19** | **0.23** | **1.21** | **0.70** | **0.88** | **24.87** | |
|  | 4 | 1000 | No | 1.09 | 0.37 | 0.33 | 0.51 | 0.84 | - | |
|  | 32 | 1000 | Yes | 0.82 | 0.32 | 0.39 | 0.77 | 0.53 | - | |
|  | 47 | 1000 | Yes | 0.96 | 0.40 | 0.41 | 0.77 | 0.26 | - | |
|  | 123 | 1000 | Yes | 0.57 | 0.18 | 0.31 | 0.71 | 0.27 | - | |
| CASE-9 | **0** | **-** | **Yes** | **0.72** | **0.76** | **1.05** | **0.83** | **0.83** | **26.37** | |
|  | 2 | 1600 | No | 0.77 | 0.59 | 0.76 | 0,82 | 0.67 | - | |
|  | 7 | 1600 | Yes | 0.73 | 0.15 | 0.20 | 0.72 | 0.39 | - | |
|  | 14 | 1600 | Yes | 0.66 | 0.11 | 0.16 | 0.74 | 0.36 | 61.95 | |
|  | 21 | 1600 | Yes | 0.66 | 0.12 | 0.18 | 0.90 | 0.27 | - | |
|  | 35 | 1600 | Yes | 0.94 | 0.12 | 0.12 | 0.87 | 0.28 | >75 | |
|  | 61 | 1600 | Yes | 0.94 | 0.12 | 0.12 | 0.86 | 0.47 | 51.41 | |
|  | 90 | 1600 | Yes | 0.82 | 0.15 | 0.18 | 0.80 | 0.31 | - | |
| Abbreviations: NA: not available; IS/P ratio: infants plasma-to-maternal plasma ratio; eGFR= estimated filtration glomerular rate (the estimated GFR values ​​were reported if they were less than 75 mL/min/1.73 m^2^, otherwise they were > 75 mL/min/1.73 m^2^)^39^.  ^†^ Technical interference  ^‡^ Insufficient sample | | | | | | | | | | |
